# Integrating bulk and single-cell RNA sequencing data reveals the relationship between intratumor microbiome signature and host metabolic heterogeneity in breast cancer

**DOI:** 10.3389/fimmu.2023.1140995

**Published:** 2023-03-14

**Authors:** Fangyue Chen, Jun Yang, Youxiang Guo, Dongwei Su, Yuan Sheng, Yanmei Wu

**Affiliations:** ^1^Department of General Surgery, Changhai Hospital, Navy Military Medical University, Shanghai, China; ^2^Department of General Surgery, 63650 Military Hospital, Urumqi, China

**Keywords:** intratumoral microbiome, breast cancer, metabolic heterogeneity, tumor microenvironment, immune cell

## Abstract

**Introduction:**

Nowadays, it has been recognized that gut microbiome can indirectly modulate cancer susceptibility or progression. However, whether intratumor microbes are parasitic, symbiotic, or merely bystanders in breast cancer is not fully understood. Microbial metabolite plays a pivotal role in the interaction of host and microbe via regulating mitochondrial and other metabolic pathways. And the relationship between tumor-resident microbiota and cancer metabolism remains an open question.

**Methods:**

1085 breast cancer patients with normalized intratumor microbial abundance data and 32 single-cell RNA sequencing samples were retrieved from public datasets. We used the gene set variation analysis to evaluate the various metabolic activities of breast cancer samples. Furthermore, we applied Scissor method to identify microbe-associated cell subpopulations from single-cell data. Then, we conducted comprehensive bioinformatic analyses to explore the association between host and microbe in breast cancer.

**Results:**

Here, we found that the metabolic status of breast cancer cells was highly plastic, and some microbial genera were significantly correlated with cancer metabolic activity. We identified two distinct clusters based on microbial abundance and tumor metabolism data. And dysregulation of the metabolic pathway was observed among different cell types. Metabolism-related microbial scores were calculated to predict overall survival in patients with breast cancer. Furthermore, the microbial abundance of the specific genus was associated with gene mutation due to possible microbe-mediated mutagenesis. The infiltrating immune cell compositions, including regulatory T cells and activated NK cells, were significantly associated with the metabolism-related intratumor microbes, as indicated in the Mantel test analysis. Moreover, the mammary metabolism-related microbes were related to T cell exclusion and response to immunotherapy.

**Conclusions:**

Overall, the exploratory study shed light on the potential role of the metabolism-related microbiome in breast cancer patients. And the novel treatment will be realized by further investigating the metabolic disturbance in host and intratumor microbial cells.

## 1 Introduction

Vertebrates co-evolve with microbes, and humans are hosts to diverse microbial symbionts (1–3). Microbiota is a non-negligible part of the human body and exerts significant effects on host metabolism, immune system, and disease progression (4, 5). The role of the microbiome in tumor initiation, prognosis, and response to therapy has been discussed for a long time. In the last few years, the relationship between cancer and microbe was mainly focused on gastrointestinal microbiota dysbiosis; however, cumulative evidence supports the concept that intratumoral microbiota is an integral component of the extra-intestinal tumor as well (6, 7).

Human breast tissue is not as sterile as previously thought, and it contains a distinct microbiota community compared with other tissues in the human body (8, 9). The breast microbiome mainly originates from the gastrointestinal tract or skin *via* nipple-areolar orifices (10). Cultivable bacteria with broad microbial viability have been found in mammary tumor tissues, and depletion of intratumor bacteria reduces breast cancer metastasis (11–13). Moreover, the complex interaction of microbiota and steroid hormone has a influence on bone metastasis of breast cancer (14).

In order to sustain rapid proliferation, cancer cells compete for more nutrients and sense metabolite alteration to coordinate biological behavior (15). Recent studies have revealed that extra- or intracellular communities of microbes are metabolically active and immunoreactive (16). And metabolic microenvironment of cancer may be regulated and reshaped by the gut or intratumor microbiome (17–19). For example, nicotinamide adenine dinucleotide (NAD) metabolism and estrogen metabolism of breast cancer are modulated by host-microbe metabolic interaction (20, 21). And the change in dietary pattern has an influence on gut microbial metabolites in breast cancer patients (22, 23). Furthermore, the co-metabolism of host-microbe changes the chemotherapeutic agent efficacy in patients (24). Collectively, the previous studies raised the possible importance of the pairwise interaction of intratumor microbes and cancer metabolism. However, the relationship between them is less described and remains to be elucidated furtherly.

Here, we investigated the relationship between intratumor microbe and cancer metabolism in silico analysis. And metabolism-related microbial signature was constructed to explore the potential prognostic value. Subsequently, we analyzed the association of breast microbiome with gene mutation and immune microenvironment. Furthermore, the response to immunotherapy and chemotherapeutics was evaluated based on microbial abundance. These findings revealed a relatively comprehensive map of intratumor metabolism-related microbes, with insight into novel breast cancer treatment. However, the available results were merely correlative in silico analysis, and experiments on isolated intratumor microbes were needed to validate these findings.

## 2 Materials and methods

### 2.1 Microbiome, bulk, and scRNA-seq data source and processing

We obtained 1085 breast cancer (BRCA) samples with normalized intratumor microbial abundance (measured in 1406 genera) from The Cancer Genome Atlas (TCGA) database. Poore et al. (25) re-examined whole genome and transcriptome sequencing studies in TCGA for microbial reads. The intratumor microbial abundance data file, Kraken-TCGA-Voom-SNM with all putative contaminants removed, was used for analysis in our study. The microbial abundance and metadata files were downloaded from the online data repository (http://ftp.microbio.me/pub/cancer_microbiome_analysis/). The matched clinical data, mutation information, and transcription matrix were acquired from TCGA datasets using TCGAbiolink package (26). The samples without complete clinical information were excluded. And the analysis of the microbe-host mutation relationship was performed based on maftools package. The matched metabolite profiling of the TCGA-BRCA cohort was acquired from previous research (27). Human breast single-cell RNA (scRNA) sequencing data was downloaded from GSE161529 dataset (28) in the Gene Expression Omnibus (GEO) database. A total of 32 scRNA-seq samples were selected for the study, including 8 triple-negative breast cancer (TNBC) and 24 non-TNBC (ER+ and HER2+) cases. Seurat (v.4.3.0) was used for data processing and quality control. The cells with unique molecular identifier count < 300 and mitochondrial content > 10% were defined as low-quality cells and excluded subsequently. Finally, a total of 154,179 single cells passed quality control. Harmony package (29) was used to integrate the scRNA-seq samples and control batch effects. Major cell types were annotated preliminarily based on gene sets from CellMarker 2.0 (30): epithelial cells (*EPCAM*, *KRT19*); myeloid cells (*CD68*, *CD163*); fibroblasts (*COL1A1,PDGFRB*, *ACTA2*); T and NK cells (*CD3D*, *CD4*, *NKG7*); plasma cells (*JCHAIN*, *IGHG1*, *MZB1*); endothelial cells (*PECAM1*, *VWF*); B cells *(CD79A*, *MS4A1*).

### 2.2 Metabolic activity evaluation and metabolism-related microbe clusters

Metabolism-related pathway gene sets were downloaded from Kyoto Encyclopedia of Genes and Genomes (KEGG) database. The various metabolic activities of TCGA-BRCA samples were estimated by the gene set variation analysis (GSVA) (31). Then, we used spearman correlation coefficient to evaluate the association between intratumor microbiota and metabolic activity in paired TCGA samples. The scMetabolism package (32) and SCPA package (33) were used to quantify and visualize the metabolic features and other pathway characteristics of breast cancer cells at single-cell resolution. Program iClusterPlus package (version 1.34.3) was developed for integrative analysis of multi-type genomic data, and we used it to generate an integrated cluster assignment based on microbial abundance and the metabolism of cancer cells.

### 2.3 Functional enrichment analysis and weighted correlation network analysis

The differentially expressed genes between the two clusters were identified using limma package. Subsequently, the Gene Ontology (GO) and the Gene Set Enrichment Analysis (GSEA) were used to investigate the potential biological function. The clusterProfiler package (34) was used for the above analysis. Weighted correlation network analysis (WGCNA) (35) was performed with default parameters. The module eigengene (ME) was used to describe the expression pattern of the modules. We screened the microbe-related modules by evaluating the correlation between WGCNA modules and microbial abundance. The module membership (MM) was used to describe the reliability of genes in modules. The hub genes were selected based on modular connectivity.

### 2.4 Using scissor algorithm to identify microbe-associated single cells

Scissor (Single-cell identification of subpopulations with bulk sample phenotype correlation) method (36) was used to identify bulk phenotype-associated cell subpopulations. Scissor cells exhibited distinct molecular properties relevant to the biological processes of the given phenotypes. In our study, the input data of Scissor pipeline consisted of GSE161529 scRNA-seq data, TCGA-BRCA expression matrix, and matched TCGA microbial abundance data. The abundance of the microbial genus was regarded as the phenotypical features of matched TCGA samples. Then, the correlation matrix was constructed to quantify the similarity between the GSE161529 single-cell data and TCGA-BRCA bulk data. Based on the signs of the estimated regression coefficients, the single cells were classified into Scissor positive (Scissor+) and negative (Scissor-) cells, which were positively and negatively associated with the matched microbial abundance, respectively. In the above processing, we set the parameter α equal to 0.8. Finally, the metabolic activities of Scissor-selected cells were further characterized in downstream analyses.

### 2.5 Construction and validation of metabolism-related microbe signature

Multivariate cox proportional hazard model was applied to assess the relative risk for metabolism-related microbial abundance. The risk scores were calculated with the formula: risk score = $\sum_{k=1}^{n}$ abundance (Microbe k) × coefficient (Microbe k). Then, the patients were divided into high- and low-risk group according to the median value of scores. In order to validate the function and predictive value of the microbial signature model, TCGA-BRCA patients were randomized into training and validation sets. The concordance index (C-index) and area under the receiver operating characteristic (ROC) curve were used to estimate the predictive accuracy of the model. A nomogram that integrated microbial scores and clinical characteristics was developed to predict the overall survival rate. And calibration curve was performed to validate the predictive performance of the nomogram.

### 2.6 Inferring aberrant pathway and analysis of immune microenvironment

PROGENy (37) algorithm was used to infer 14 signaling and regulatory pathways of breast cancer: androgen, EGFR, estrogen, hypoxia, JAK-STAT, MAPK, NF-kB, p53, PI3K, TGF-b, TNF-a, Trail, VEGF, and WNT. We used CIBERSORT (38) algorithm to analyze differences in the proportion level of immune cells within breast cancer tissues. We applied Mantel test to explore the correlation between metabolism-related microbiota at the phylum level and immune cell infiltration of tumor. Repressed immune resistance (RIR) is a malignant cell program associated with T cell exclusion (39). We used EaSIeR (40) package to calculate RIR scores (resF_down, resF_up, resF), and the immune checkpoint inhibitor resistance was predicted according to the obtained data.

### 2.7 Predicting response to chemotherapeutic agents

The OncoPredict (41) package was used to evaluate the semi-inhibitory concentration (IC50) of drugs in GDSC database based on bulk gene expression data. Then, we investigated the relationship between metabolism-related microbial abundance and drug sensitivity.

### 2.8 Statistical analysis

All graphical representations and statistical analyses were performed based on R software (version 4.2.0), and all methods mentioned in the study were available as R packages. Overall survival (OS) rates were calculated by Kaplan-Meier method, and difference in survival rates was assessed by log-rank test. Nonparametric comparisons in the study were performed using Wilcoxon test.

## 3 Results

### 3.1 Relationship between intratumor microbial abundance and metabolic activity

**Figure 1:**
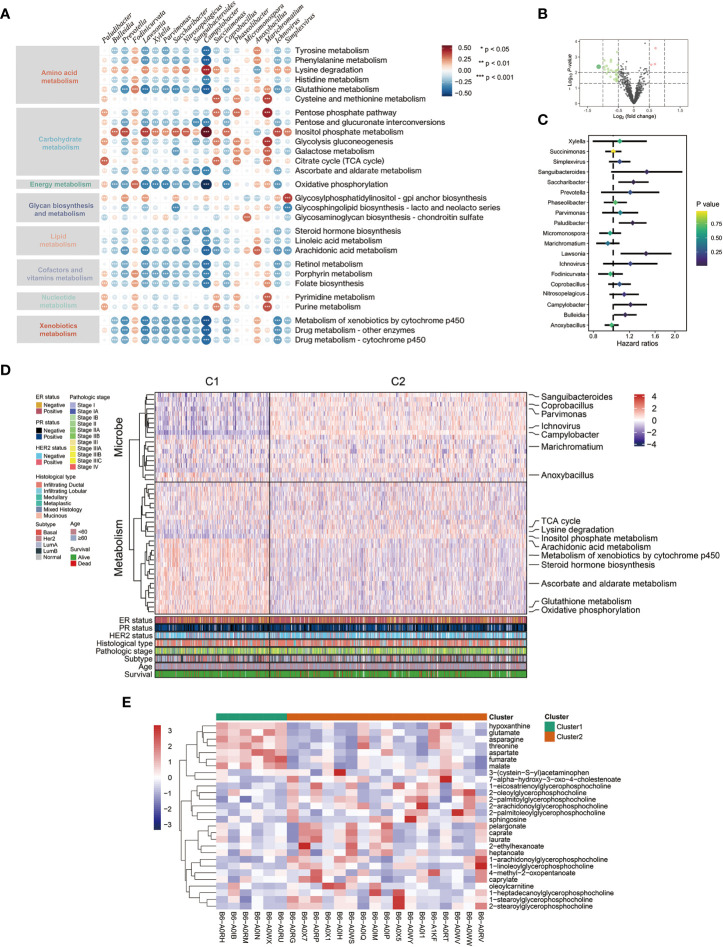
Relationship between intratumor microbe and metabolic pathway in breast cancer patients. **(A)** Spearman correlation between metabolism activity and microbial abundance in TCGA-BRCA dataset (red: positive relevant; blue: negative relevant). **(B)** Volcano plot displaying microbial abundance difference between breast normal and tumor tissue. **(C)** Forest plot of prognosis value of metabolism-related microbes from TCGA-BRCA dataset. **(D)** Integration of microbe and metabolism data defined two clusters using iClusterPlus package. **(E)** The heatmap showing significant differential metabolites between the two clusters.

**Figure 2:**
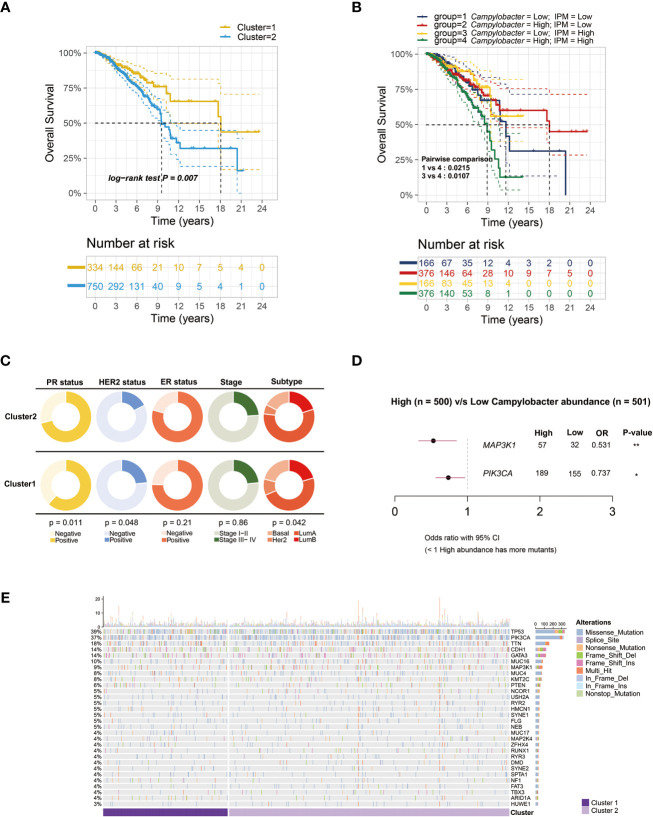
Survival analysis and microbe-related mutation landscape. **(A)** Kaplan-Meier overall survival plots between the two clusters based on microbial abundance and metabolic activity (P = 0.007). **(B)** Kaplan-Meier overall survival plots of breast cancer patients separated into four groups by *Campylobacter* abundance and inositol phosphate metabolism (IPM) activity (P = 0.0107). **(C)** Pie charts showing the Chi-squared test of clinicopathologic factors between the two clusters in TCGA-BRCA samples. **(D)** Forest plot of different genes mutation between low and high *Campylobacter* abundance group. **(E)** The waterfall plot illustrating the top 30 most common somatic mutant genes in the two clusters.

Firstly, we calculated GSVA scores to evaluate the activities of various metabolic pathways in TCGA-BRCA samples. Then, the relationship between intratumor microbiota and metabolic activity was analyzed. The microbes, whose correlation coefficient (|r|) > 0.3 and P < 0.001, were regarded as metabolism-related. Under this criterion, 19 genera of microbes, including *Lawsonia, Bulleidia, Campylobacter*, and so on, were significantly correlated with the activities of 8 major metabolic pathways (**Figure 1A**). Additionally, a volcano plot showed that the microbiota present in tumor tissue was different from mammary tissue (**Figure 1B**). Univariate survival analysis of metabolism-related microbes showed that *Campylobacter*, *Saccharibacter*, *Paludibacter*, *Lawsonia*, and *Bulleidia* were prognostic factors in breast cancer patients (**Figure 1C**). Then, TCGA-BCRA patients were divided into two clusters (C1 and C2) using iClusterPlus method based on intratumor microbial abundance and activity of metabolic pathways. The differences in microbial abundance and heterogeneous metabolic activity between the two clusters are depicted in **Figure 1D**. The distribution of metabolites between cluster 1 and cluster 2 was analyzed using matched metabolomics data from the previous study. And the metabolites whose P values less than 0.05 are demonstrated in the heatmap (**Figure 1E**). Kaplan-Meier survival curve plot showed that cluster 2 patients had lower survival probability (**Figure 2A**). The chi-squared test showed that the two clusters exhibited different status of PR, HER2, and molecular subtype (**Figure 2C**).

### 3.2 Biological function enrichment analysis and WGCNA

**Figure 3:**
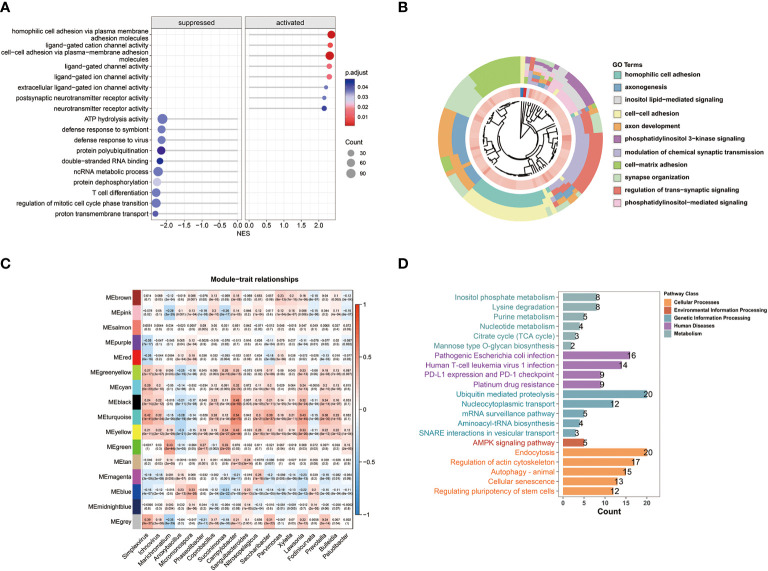
WGCNA and biological function analysis. **(A)** Gene set enrichment analysis (GSEA) and normalized enrichment scores (NES) between the two clusters. **(B)** Biological process function enrichment from Gene Ontology (GO) analysis of the two clusters. **(C)** WGCNA of breast cancer samples showing module eigengenes (ME) that were correlated with metabolism-related microbial abundance. **(D)** Results of KEGG pathway enrichment analysis for the genes in WGCNA black and turquoise modules.

The GSEA results demonstrated that C2 patients with relatively high microbial abundance had suppressed defense response to symbiont. On the other hand, cell adhesion and ligand-gated ion channel activity were activated in C2 patients (**Figure 3A**). Moreover, the GO analysis further revealed that the cluster-related signal pathways included regulation of cell adhesion, inositol phosphate metabolism, T cell differentiation, etc. (**Figure 3B**). Subsequently, we constructed a co-expression network in TCGA-BRCA samples by weighted gene co-expression network analysis (WGCNA). In the analysis, we treated the abundance of metabolism-related microbes as the phenotype of the samples. The minimum module size for module detection was set to 50, and 16 modules were obtained from WGCNA. The figures that emerged from WGCNA analysis are presented in **Figure S1**. Then, black and turquoise modules, which were most relevant to microbial abundance, were selected for the subsequent analysis (**Figure 3C**). In order to explore biological function, pathway enrichment analysis of genes in two modules was performed based on KEGG database. The biological categories and top enriched pathways, including inositol phosphate metabolism, nucleotide metabolism, pathogenic *Escherichia coli* infection, and drug resistance, are demonstrated in **Figure 3D**. Given the high degree of correlation between intratumor *Campylobacter* abundance and inositol phosphate metabolism, their prognostic values were explored in breast cancer patients. Survival analysis revealed that patients with both high *Campylobacter* abundance and inositol phosphate metabolic activity had the worst survival probability (**Figure 2B**).

### 3.3 Association between mutation landscape and metabolism-related microbe

We collected the matched somatic mutation data to investigate the mechanism of metabolism-related microbe in breast cancer. Strikingly, the results indicated that specific microbial abundance might play a key role in tumorigenesis or tumor progression. We found that *MAP3K1* and *PIK3CA* were more frequently mutated in samples with higher intratumoral *Campylobacter* abundance (**Figure 2D**). The mutation genes between the two clusters are depicted in **Figure 2E**. And there was no significant difference between the two clusters in the ratio of non-synonymous and synonymous mutation.

### 3.4 Quantifying metabolic activity at single-cell resolution

**Figure 4:**
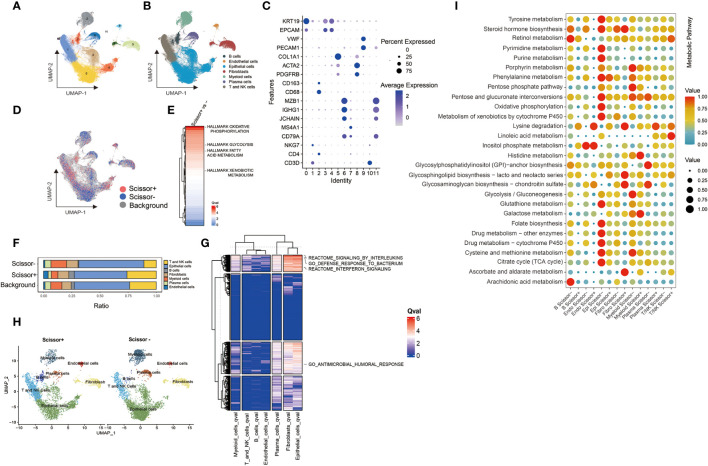
Results of Scissor algorithm and metabolic activity at single cell resolution. **(A, B)** UMAP visualization of cell clusters and cell-type-specific annotation in scRNA-seq dataset. **(C)** Visualization of gene expression using dot plot. **(D)** UMAP visualization of Scissor+ and Scissor- cells. The red and blue dots were the cells associated with selected microbial phenotype. **(E)** Ranking of Qvals of Hallmark pathway in Scissor+/- cells. **(F)** Proportional fractions of identified cell types across Scissor+/- condition. **(G)** Ranking of Qvals of Hallmark, Reactome and GO pathway in Scissor+/- cells with various cell types. **(H)** UMAP representations of identified cell types across Scissor cells. **(I)** The metabolic pathway activity in different cell types in breast cancer scRNA-seq dataset.

We used the standard Seurat pipeline to analyze single-cell RNA (scRNA) sequencing data. Following dimension reduction, cell clustering, and annotation of cell type, the cells were grouped into 7 types, including 35,940 T and NK cells, 4,120 B cells, 15,291 myeloid cells, 76,147 epithelial cells, 2,088 endothelial cells, 7,703 plasma cells, and 12,890 fibroblasts (**Figures 4A–C**, **S2**). Considering the high degree of relevance between *Campylobacter* and WGCNA module eigengene (ME), the abundance of this genus in TCGA-BRCA was typically selected as the phenotype to identify the most highly associated cell subpopulations from scRNA-seq data. Then, 4,621 Scissor+ cells and 5,082 Scissor- cells were classified by the microbial phenotype of bulk samples (**Figure 4D**). Proportion of cell types showed that proportional fractions of T/NK and B cells in Scissor+ group were higher than Scissor- group (**Figures 4F, H**). We plotted the heatmaps of the qvals to demonstrate broad patterns (Hallmark, Reactome, and GO pathways) between Scissor+/- cell subpopulations. And we highlighted metabolic pathways, including glycolysis, fatty acid metabolism, and xenobiotic metabolism, in **Figure 4E**. Besides, the dysregulation of pathways, including response to bacterium, antimicrobial humoral response, interferon, and interleukins signaling, were observed in Scissor+/- cells, suggesting the identified cells were associated with tumor-resident microbes (**Figure 4G**). Furthermore, metabolic analysis of scissor-selected cells among different cell types was performed using scMetabolism method. And activities of the metabolic pathways in Scissor+/- cells were assessed as depicted in **Figure 4I**. We found that metabolic differences in single cells were similarly compared with bulk samples.

### 3.5 Establishment and validation of metabolism-related microbial model

**Figure 5:**
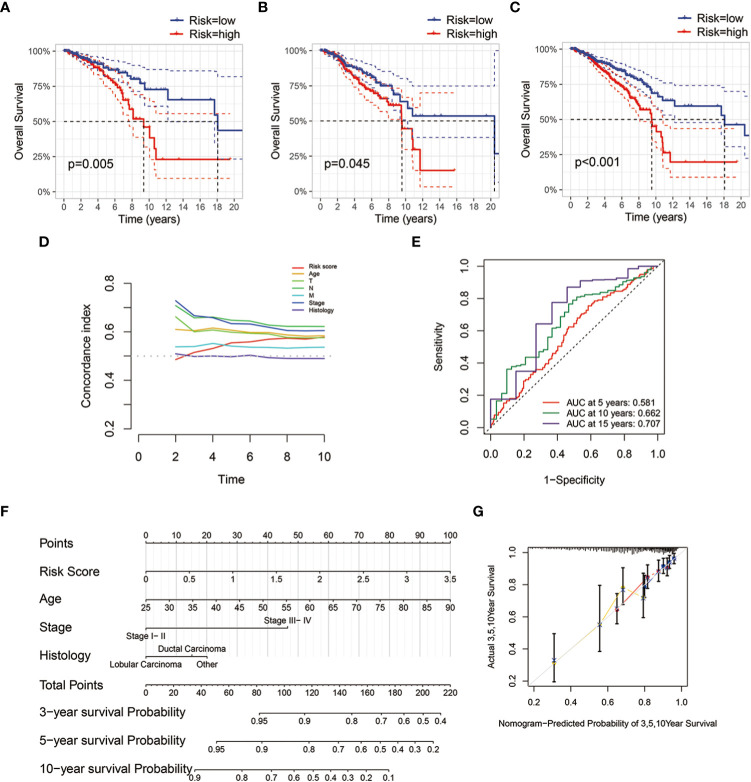
Establishment and validation of microbial risk model and nomogram. **(A–C)** Kaplan-Meier survival plots of the microbial risk groups in the training, validation and full data sets. **(D)** Distributions of concordance index (c-index) values of TCGA-BRCA microbial risk model. **(E)** The 5-, 10- and 15-year ROC curves of breast cancer patients with low or high microbial risk scores. **(F)** Nomogram with microbial risk scores and clinicopathologic characteristics for predicting overall survival of breast cancer patients in TCGA cohort. **(G)** Calibration curve of overall survival nomogram model.

We randomly split the TCGA-BRCA patients into two groups, training and validation sets. We evaluated risk factors using the multivariate Cox proportional hazard model based on metabolism-related microbial abundance. The risk score = (*Campylobacter*×0.328) + *(Paludibacter*×0.285) + (*Marichromatium*×-0.194). The patients were divided into low- and high-risk groups using the median cutoff value of risk scores. With regard to overall survival, patients with high-risk scores had a significantly poorer prognosis in the training, validation, and full data sets (**Figures 5A–C**). The concordance index is demonstrated in **Figure 5D**. The 10-year AUC value of the model was 0.662, indicating accurate predictive performance in long-term outcome (**Figure 5E**). A nomogram was established to predict the overall survival probability based on microbial scores and other pathological features (**Figure 5F**). The calibration plot of the nomogram showed high consistency between prediction and actual observation (**Figure 5G**).

### 3.6 Aberrant pathway signaling and immune microenvironment associated with metabolism-related microbiome

**Figure 6:**
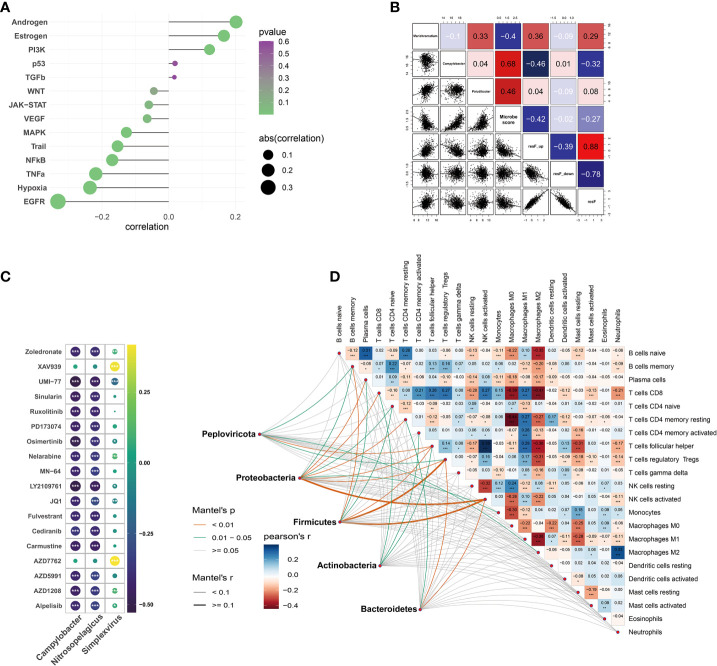
Tumor immune microenvironment and correlation between drug sensitivity and microbial abundance. **(A)** Correlation between pathway activity estimated by PROGENy algorithm and metabolism-related microbial scores. **(B)** Correlations between microbiome and repressed immune resistance (RIR) scores (Blue color represents the negative correlation; the red represents the positive correlation). **(C)** Analysis of drug sensitivity associated with host metabolism-related microbiome (correlation coefficient (|r|) > 0.4). **(D)** Mantel test on the metabolism-related microbes at phylum level and CIBERSORT immune cell fractions in matched samples. *p < 0.05, **p < 0.01, ***p < 0.001.

PROGENy analysis showed that microbial scores were associated with pathway perturbation, including EGFR, estrogen, androgen, hypoxia, and so forth (**Figure 6A**). Repressed immune resistance (RIR) is a predictive biomarker for immune checkpoint inhibitors response. The *Campylobacter* abundance and microbial scores were negatively associated with resF_up and resF score, suggesting the metabolism-related microbes were involved in immunotherapy resistance (**Figure 6B**). In order to determine whether microbes were related to the breast immune microenvironment, we evaluated the association between microbial scores and 22 types of immune cells. The results of Mantel test showed the metabolism-related microbes at the phylum level were significantly correlated with memory B cells, resting memory CD4+ T cells, T follicular helper cells, regulatory T cells, and activated NK cells (**Figure 6D**). These results suggested that intratumoral microbiota was linked with the heterogeneity of the host immune microenvironment.

### 3.7 Microbe-related drug sensitivity prediction

OncoPredict package was utilized to predict the potential response to chemotherapy based on microbial abundance. The absolute value of the drug-microbe correlation coefficient greater than 0.4 is demonstrated in **Figure 6C**. Metabolism-related microbial genera, notably *Campylobacter*, *Nitrosopelagicus*, and *Simplexvirus*, were associated with drug sensitivity of Zoledronate, Alpelisib, Fulvestrant, Nelarabine, etc.

## 4 Discussion

Intratumor microbiome is an emerging field of cancer research, and it has been studied over the past few years. Numerous species and strains of microbes with unique patterns have been identified in certain tumor types (17). For instance, changed saliva carriage of microbe, such as *Bulleidia*, is a reliable indicator of clinical disease progression in patients with esophageal cancer (42). And pancreatic cancer tissue had an altered intratumoral microbiome profile with decreased *Bacteroidales* and increased *Campylobacterales* (43). Moreover, increased *Campylobacter* abundance in gut microbiota is a risk factor for the occurrence of breast cancer (14). Microbiota and host interact bidirectionally in many ways. The extra- and intracellular microbes have effects on tumorigenesis and cancer progression in the aspects of genome, transcriptome, and metabolome (44, 45). It is worth mentioning that increasing nutrient within the area of tumor necrosis is conducive to bacteria survival. Furthermore, microbial metabolites modulate the biological behavior of breast cancer and reshape the tumor microenvironment (46, 47).

The relationship between intratumor microbe and metabolism was depicted comprehensively in our study. A total of 19 microbial genera were significantly correlated with multiple major metabolic signals. The patients with breast cancer were divided into two clusters with different survival conditions based on the tumor microbe and metabolism data. Besides, the differential analysis of matched metabolite concentration showed that intratumor microbes were closely relevant to metabolite disorder. The GSEA analysis supported the biological significance that the defense response to symbiont was different between the two clusters. Then, KEGG and GO enrichment analyses were performed to investigate the biological processes involved in the microbe existence. The most significant pathways consisted of environment information processing, genetic information processing, and metabolism. It was noteworthy that inositol phosphate metabolism was enriched remarkably in both KEGG and GO analysis. Furthermore, Kaplan-Meier survival analysis showed that patients with both high *Campylobacter* abundance and inositol phosphate metabolic activity had the worst prognosis. The previous mass spectrometry analyses indicated that inositol phosphate metabolism was one of the most impacted metabolic pathways in early breast cancer patients (48). And a recent study showed that microbiota-derived inositol trisphosphate could promote epithelial reparation by activating mammalian histone deacetylase (49), which reflected biological significance.

The intratumor microbes were distributed heterogeneously across tumors at the microscopic level (50). We used Scissor algorithm to make the bulk data with matched microbial abundance mapped into the scRNA-seq dataset. Given the highest correlation between *Campylobacter* and tumor metabolism, this bacterial genus was chosen as the phenotype to identify the associated cell subpopulations. The metabolic abnormality and heterogeneity were quantified at single-cell resolution by using scMetabolism package to evaluate scissor+/- cells. Overall, the results of bulk and scRNA-seq data demonstrated that intratumor microbes were associated with host intracellular metabolic reprogramming.

Analysis of gene mutation showed that patients with high intratumor *Campylobacter* abundance were characterized by the increased mutation frequency of *MAP3K1* and *PIK3CA*. The possible mechanism may be the microbe-mediated failure of double-strand DNA break repair and consequent genomic instability (51). And it has been found that microbes could cause genotoxin-mediated mutagenesis in colorectal and urinary tract cancers (52, 53). Taken together, the breast metabolism-related intratumor microbial pattern may have influence on host specific genetic alterations. The bacteria invading host mammary cells turned on various aberrant signals in the host, such as TNF signaling pathway, NF-kB signaling pathway, and the cytokine chemokine related pathway (13). And aberrant pathway signaling was also observed in our study. These aberrant signals may promote or initiate the development of breast cancer.

In addition to host metabolism, microbes had profound effects on the modulation of the tumor immune microenvironment (54–56). We performed Mantel test to investigate the extensive crosstalk between infiltrating immune cells and metabolism-related microbes at the phylum level. Among all infiltrating immune cells, regulatory T cells and activated NK cells were mostly correlated with microbial abundance. Previous studies revealed that local microbiota provoked lung adenocarcinoma-associated inflammation by activating lung-resident T cells (57). And *Lactococcus* activated natural killer T cell to promote cellular immunity in breast cancer (58). Besides, bacteria activated anti-tumor immune response through specific antigens. For example, Bacillus Calmette-Guerin (BCG) was used as immunotherapy in bladder cancer (59). In recent years, immunotherapy attracted a great deal of attention for its success in treating solid tumors. The alternation of human commensal microbiota, such as *Bifidobacteria* and *Bacteroides fragilis*, induced anti-tumor immunity and potentiated efficacy of anti-PD-L1 or CTLA-4 blockade (60, 61). The repressed immune resistance (RIR) scores were negatively associated with microbial abundance and risk scores in our study. In this regard, the mammary intratumor microbes may be involved in immunotherapy resistance. Although the observations above depicted the intratumor microbial immune landscape, an exact causal relationship was not unambiguously established and remained elusive.

As for chemotherapy, a study revealed that dysbiosis of intratumor bacteria might contribute to gemcitabine resistance in ductal adenocarcinoma (45). We used oncoPredict package to predict the TCGA-BRCA patients’ response to a large number of drugs screened in GDSC database. Metabolism-related microbial abundance was highly correlated with drug sensitivity of Alpelisib (PI3K inhibitor), Fulvestrant (selective ER down-regulator), Nelarabine (DNA synthesis inhibitor), etc. And the potential therapeutic agents may be selected when the IC50 value was lowest among distinct clusters.

In general, our study revealed the correlation between the metabolic activity of cancer cells and the alteration of intratumor microbiota. Our analysis of metabolism-related microbes indicated their potential roles in the prognosis value and tumor immune microenvironment. Besides, microbial functions that enabled or abolished chemo- and immune therapy highlighted the potential value of correcting microbial dysbiosis in cancer treatment. However, the causal relationship is yet to be clarified in our study, and the robustness of the predictive model is limiting due to its retrospective nature. Further studies are awaited to explore how the intratumor microbes interact with the host metabolism and immune system.

## Data availability statement

The datasets presented in this study can be found in online repositories. The names of the repository/repositories and accession number(s) can be found in the article/**Supplementary Material**.

## Author contributions

FC and YW contributed to conception and design of the study. JY prepared figures and wrote the first draft of the manuscript. YG performed the statistical analysis. YS and DS wrote sections of the manuscript. All authors contributed to the article and approved the submitted version.

## Supplementary material

Supplementary Figure 1 Weighted gene co-expression network analysis. **(A)** The heatmap of microbial traits and samples dendrograms identified by WGCNA. **(B, C)** Analysis of the scale-free index and mean connectivity for various soft-threshold powers. **(D, E)** Scatterplot of gene significance versus module membership in black and turquoise modules. **(F)** Heatmap plot of topological overlap in the gene network.

Supplementary Figure 2 Visualization of marker genes on dimensional reduction plots.
