# Supplementary figures and images for: Integrating bulk and single-cell RNA sequencing data reveals the relationship between intratumor microbiome signature and host metabolic heterogeneity in breast cancer
Source: Front Immunol. 2023 Mar 14;14:1140995. doi: 10.3389/fimmu.2023.1140995 (PMC10049788; doi:10.3389/fimmu.2023.1140995)

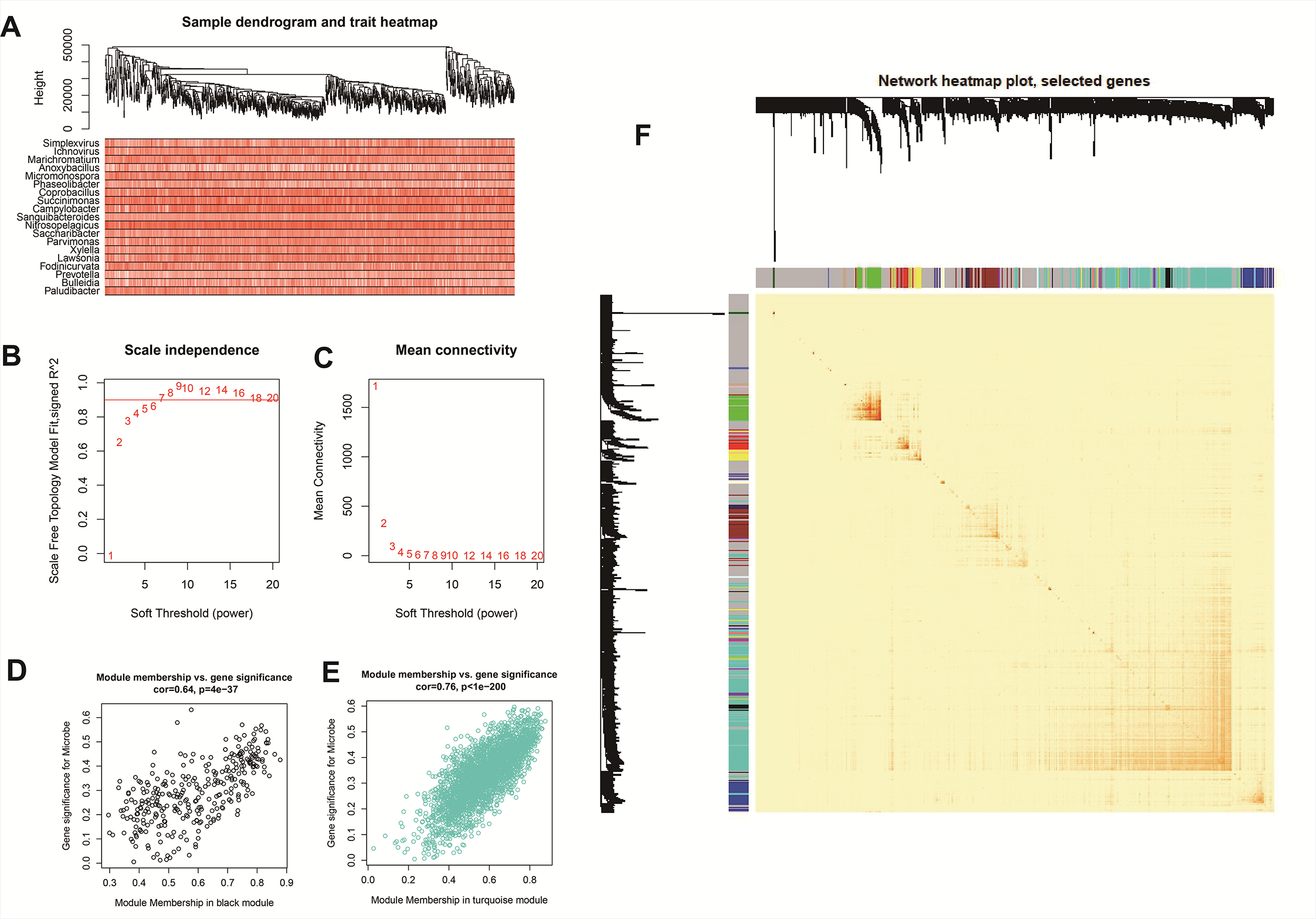

Supplement: Supplementary Figure 1 — Weighted gene co-expression network analysis. (A) The heatmap of microbial traits and samples dendrograms identified by WGCNA. (B, C) Analysis of the scale-free index and mean connectivity for various soft-threshold powers. (D, E) Scatterplot of gene significance versus module membership in black and turquoise modules. (F) Heatmap plot of topological overlap in the gene network. [file Image_1.tif]

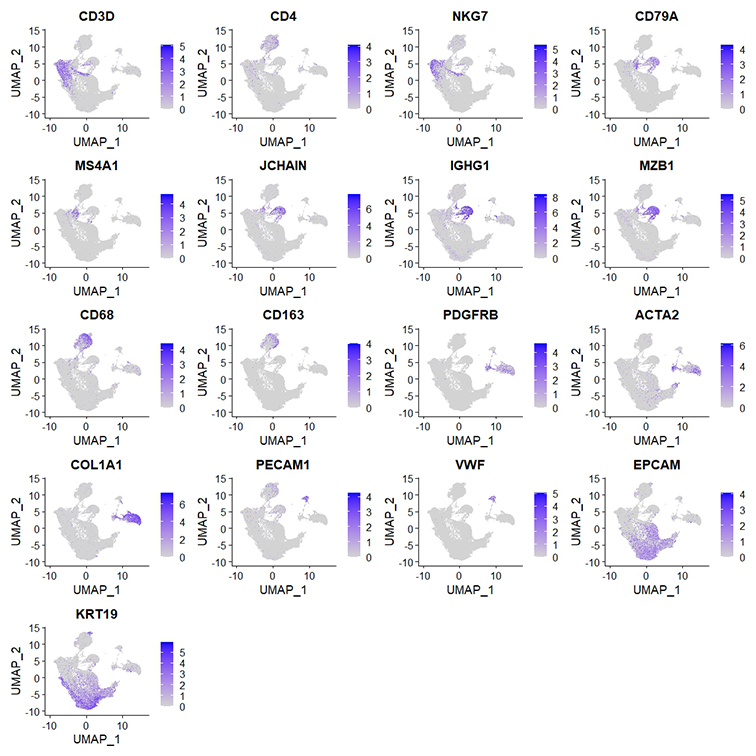

Supplement: Supplementary Figure 2 — Visualization of marker genes on dimensional reduction plots. [file Image_2.jpeg]
